# Robotic Minimally Invasive Surgery for Frontal Convexity Meningioma: A Case Report and Technical Note

**DOI:** 10.7759/cureus.112373

**Published:** 2026-07-09

**Authors:** Marco A Baralt Ramirez, Adriel Lopez Salazar, Floricel Olimpia Villegas Amador, Gianfranco Stefanoni, Ashanti Gutiérrez Quintanar

**Affiliations:** 1 Neurosurgery, Regional General Hospital 1 of the Mexican Social Security Institute, Querétaro, MEX; 2 Neurosurgery, IntegraMedica, Santiago de Chile, CHL; 3 Surgery, Regional General Hospital 1 of the Mexican Social Security Institute, Querétaro, MEX; 4 Pathological Anatomy, Regional General Hospital 1 of the Mexican Social Security Institute, Querétaro, MEX

**Keywords:** frontal meningioma, meningioma resection, neuronavigation guidance, new technologies in neurosurgery, robot-assisted surgery

## Abstract

Minimally invasive robotic-assisted cranial neurosurgery represents an evolving paradigm in the surgical management of intracranial tumors. The integration of robotic exoscopic platforms into neurosurgical workflows has demonstrated potential benefits in operative ergonomics, visualization, and microsurgical precision. Frontal convexity meningiomas, among the most common supratentorial extra-axial neoplasms, may represent an ideal candidate lesion for this approach given their superficial location and well-defined arachnoid planes. We present the case of a 23-year-old man who presented after a sports-related head injury complicated by loss of consciousness and a generalized tonic-clonic seizure. Neuroimaging revealed a well-circumscribed, homogeneously enhancing left frontal convexity lesion measuring 2.50 × 3.0 cm, with perilesional edema and a dural tail, consistent with meningioma. A neuronavigation-guided robot-assisted minimally invasive craniotomy was performed using the RoboticScope exoscope (BHS Technologies, Innsbruck, Austria) through a 4 cm incision and a 3 cm diameter craniotomy. Microsurgical subarachnoid dissection enabled circumferential tumor delineation with preservation of cortical anatomy and superior sagittal sinus venous drainage, achieving Simpson grade I gross total resection. Histopathology confirmed World Health Organization (WHO) grade I fibrous meningioma. This case demonstrates that robot-assisted minimally invasive craniotomy using a robotic exoscope system is a safe and effective approach for frontal convexity meningiomas, offering enhanced visualization, reduced brain retraction, and favorable surgical outcomes. Further prospective studies are warranted to establish standardized protocols for robotic exoscopic platforms in minimally invasive cranial neurosurgery.

## Introduction

Minimally invasive cranial neurosurgery has evolved with the goal of reducing approach-related morbidity while maintaining adequate surgical exposure and oncological control [1]. Neurosurgery has traditionally incorporated emerging technologies to improve surgical safety, precision, and efficiency, and robotic systems represent one of the most relevant recent developments in this field [2]. The keyhole concept further supports the principle of limiting surgical trauma through smaller exposures, reduced exploration of normal tissue, and less brain retraction when appropriate patient selection is applied [3].

Neurosurgical robots are classified into three categories based on surgeon-robot interaction, autonomous systems, master-slave systems, and shared control systems, each contributing to high efficiency, precision, and minimally invasive approaches [4]. Robotic exoscopic platforms offer dynamic hands-free control of magnification, focus, illumination, and camera positioning through surgeon-guided movements or voice-controlled interfaces, enabling continuous optimization of the surgical field without interrupting operative workflow. These characteristics contribute to improved operative ergonomics and may enhance microsurgical precision during minimally invasive cranial approaches [5].

Frontal convexity meningiomas are common superficial extra-axial tumors and may be suitable for minimally invasive approaches in selected cases because of their accessible location and well-defined arachnoid planes [6-8]. Clinical presentation depends on tumor size, edema, cortical irritation, and involvement of adjacent eloquent regions; seizures are particularly relevant in convexity meningiomas due to their relationship with the cortical surface [9,10]. We present a case of a left frontal convexity meningioma treated through a neuronavigation-guided minimally invasive craniotomy using a robotic exoscope system.

## Case presentation

A 23-year-old right-handed man with no significant past medical history was brought to the emergency department after sustaining a head injury during a soccer match. According to witnesses, the patient experienced a direct cranial collision with another player, immediately followed by loss of consciousness. Subsequently, he developed an apparent generalized tonic-clonic seizure. Upon arrival at the hospital, the patient underwent immediate neurological assessment and stabilization for suspected traumatic brain injury associated with post-traumatic seizure activity.

On neurological examination, the following findings were documented: Glasgow Coma Scale score of 15/15 and a Karnofsky Performance Status of 100/100. Pupils were equal, round, and reactive to light.

**Figure 1:**
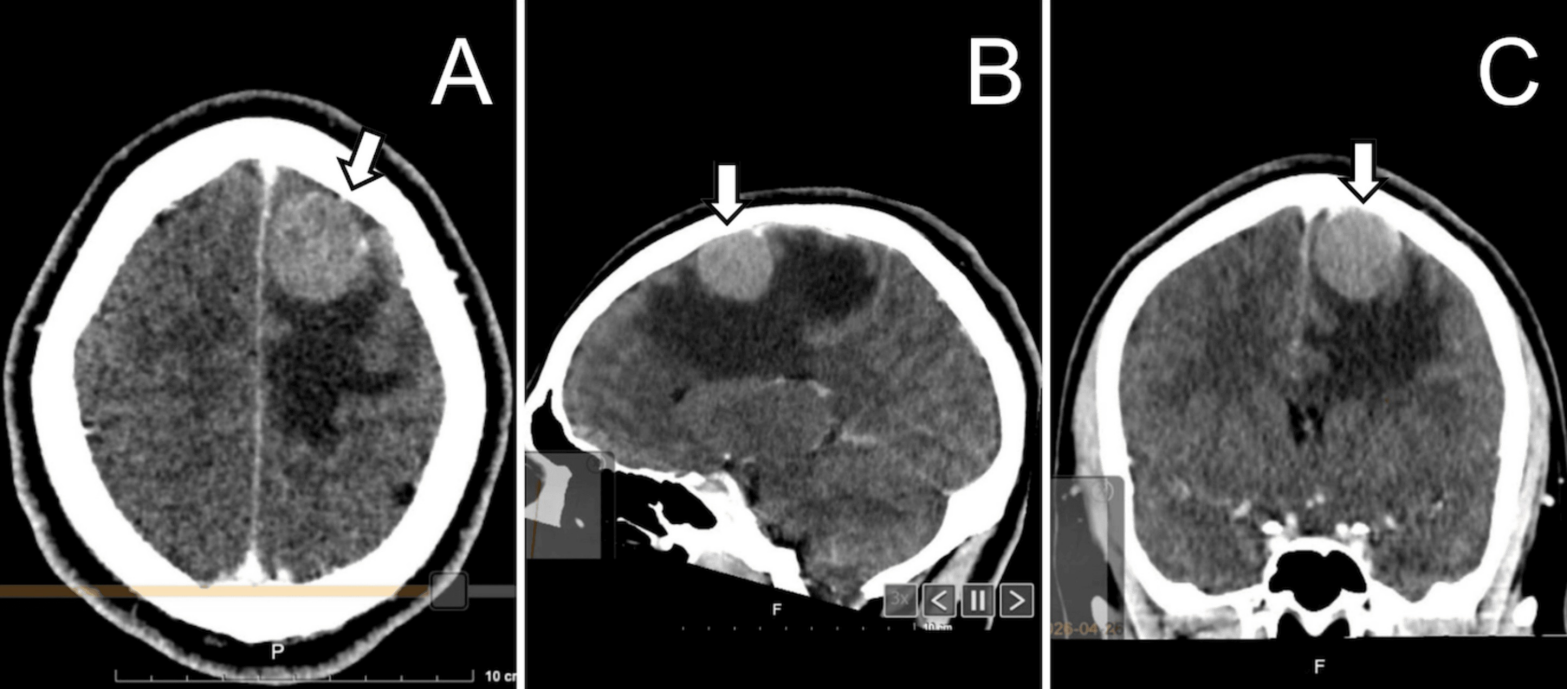
Preoperative brain CT demonstrating a left frontal convexity meningioma (A) Axial, (B) sagittal, and (C) coronal non-contrast brain CT images demonstrating a well-defined, rounded left frontal convexity mass (arrows), measuring approximately 2.5 x 3.0 cm, located within the anterior third of the superior frontal gyrus. The lesion appeared homogeneous in morphology and was associated with surrounding perilesional edema, findings suggestive of a convexity meningioma. CT: computed tomography

On computed tomography (CT), an iso- to slightly hyperdense, homogeneous, well-circumscribed, round left frontal lesion was identified, located in the left anterior third of the superior frontal gyrus, with perilesional hypodensity (surrounding lesion edema). On CT angiography, the lesion demonstrated afferent vascular supply arising from the choroidal arteries, with partial venous drainage through efferent vessels into the internal cerebral vein. The lesion measured 2.50 × 3.0 cm. No significant mass effect was observed, and there were no signs of hydrocephalus (Figure 1).

**Figure 2:**
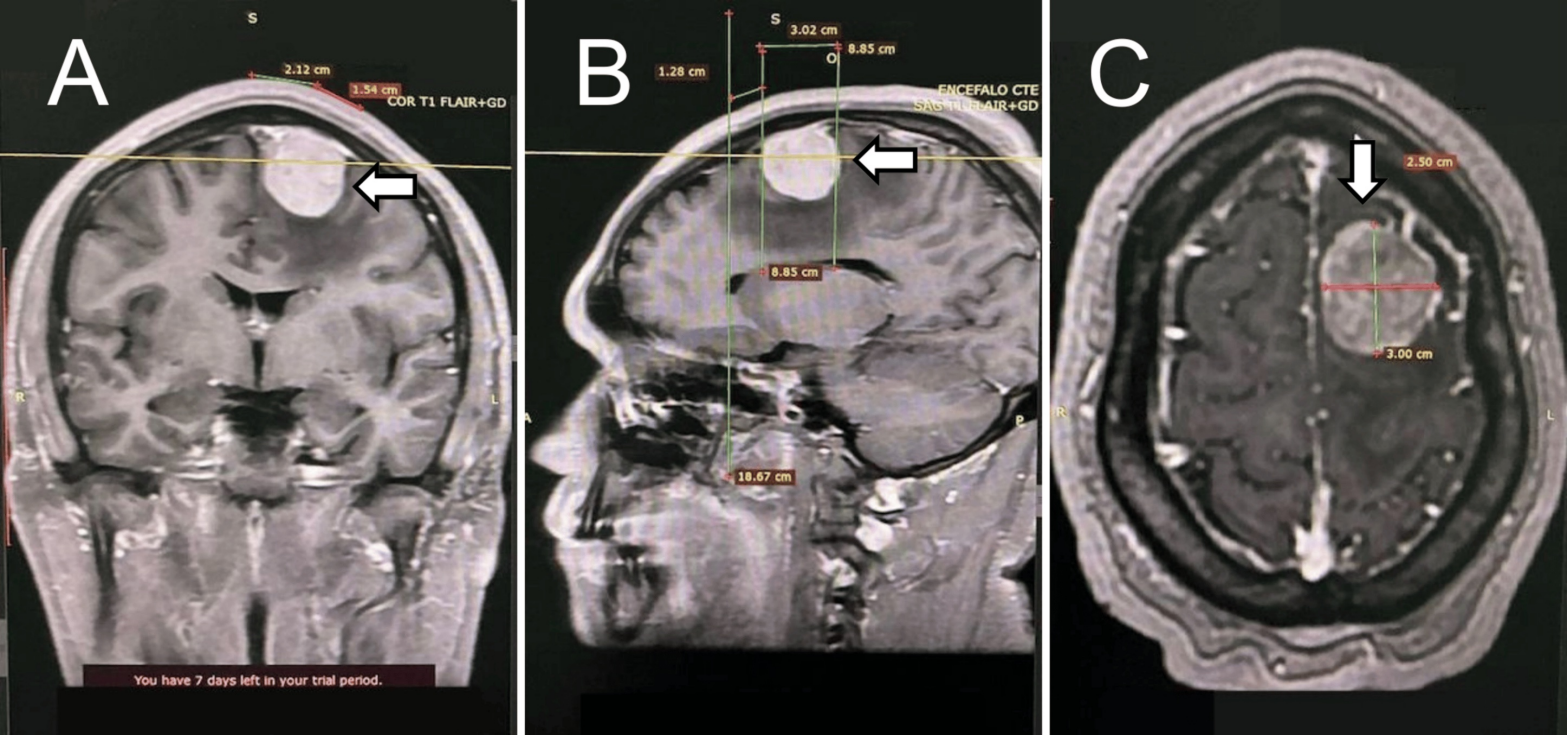
Preoperative contrast-enhanced MRI of a left frontal convexity mass (A) Coronal, (B) sagittal, and (C) axial contrast-enhanced MRI demonstrating an iso- to hyperintense, homogeneous, well-circumscribed, round lesion located at the left frontal convexity, with homogeneous contrast enhancement, surrounding perilesional edema, and a dural tail sign. MRI: magnetic resonance imaging

**Figure 3:**
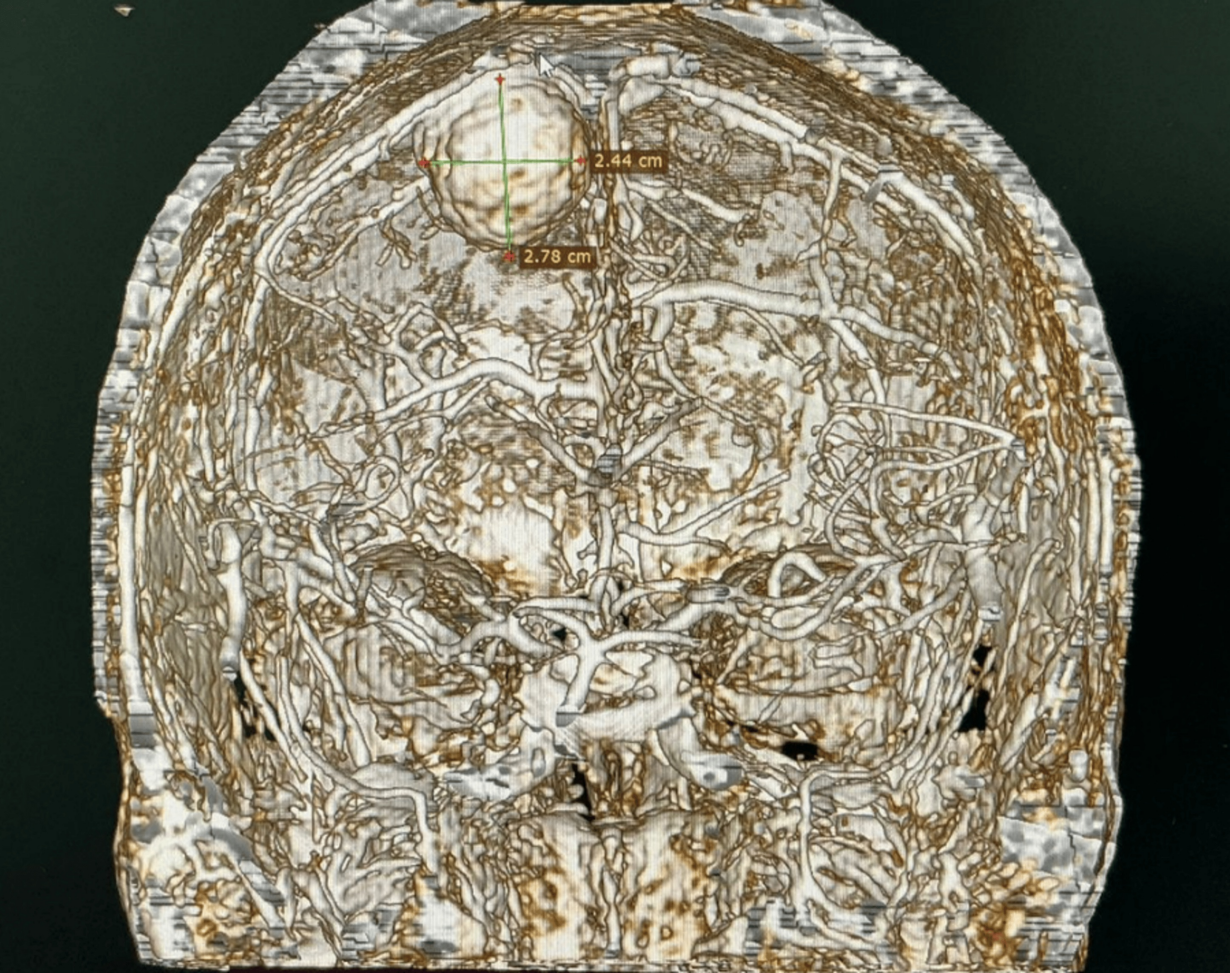
Three-dimensional vascular reconstruction Three-dimensional vascular reconstruction image demonstrating the complex vascular network surrounding the frontal convexity lesion.

Contrast-enhanced magnetic resonance imaging (MRI) confirmed a well-defined, non-infiltrative lesion with full homogeneous enhancement following contrast administration. No significant signal changes were observed on fluid-attenuated inversion recovery (FLAIR) or T2-weighted sequences. There was evidence of perilesional edema, with presence of a dural tail based on the frontal convexity (Figure 2). On reconstructed vascular imaging (Figure 3) from contrast-enhanced magnetic resonance imaging (MRI), the tumor vascular network is visualized, demonstrating an important venous drainage via a pedicle from frontal branches draining the tumor to the anterior third of the anterior longitudinal superior dural sinus.

Surgical technique

Under balanced general anesthesia, the patient was placed in the supine position with the head centered. The head was secured using a three-point fixation system with a Mayfield head clamp.

**Figure 4:**
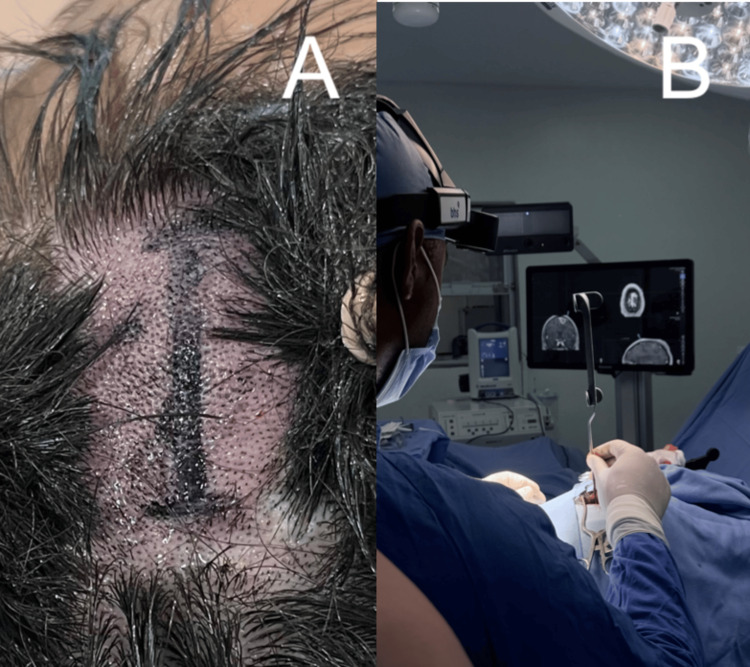
Minimally invasive frontal approach and neuronavigation planning (A) Preoperative marking of a 4 cm minimally invasive frontal incision with preservation of the patient's hair. (B) Intraoperative neuronavigation confirming the planned minimally invasive frontal entry point and surgical trajectory.

Neuronavigation was used for surgical planning to precisely define a direct tumor demarkation, limiting tumor exposure. The lesion was located 13.8 cm from the nasion at its anterior border, and the posterior border was 3.08 cm from the anterior border, in the anterior third of the superior frontal gyrus and 3 cm laterally to the midline (Figure 4).

**Figure 5:**
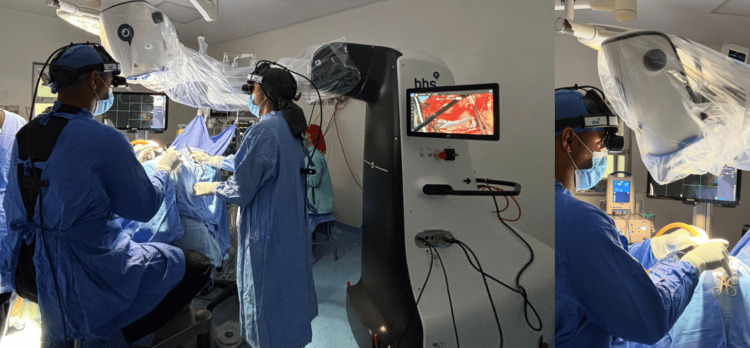
Robotic exoscope-assisted microsurgery RoboticScope exoscope (BHS Technologies, Innsbruck, Austria) provided optimal surgical positioning, enhanced visualization, and improved operative ergonomics. Dynamic adjustments of zoom, focus, illumination, brightness, and viewing angle were achieved through surgeon-controlled head movements, allowing seamless intraoperative visualization and precise microsurgical manipulation.

A linear skin incision of approximately 4 cm was made in the left frontal region. Subsequently, a minimal craniotomy measuring 3 cm in diameter was performed, centered on the previously planned entry point, enabling a minimally invasive frontal approach. Under RoboticScope exoscope (BHS Technologies, Innsbruck, Austria) (Figure 5) magnification, careful subarachnoid dissection around the tumor through the sulcus was carried out to access the 360-degree axis of the tumor.

Following an arciform dural opening with a midline-based flap, microsurgical subarachnoid dissection was performed under visualization using the BHS Technologies RoboticScope exoscope. Careful dissection allowed clear delineation of the tumor from the surrounding sulci and prominent frontal venous drainage while preserving cortical anatomy and minimizing brain retraction. This technique enabled the creation of a circumferential 360-degree surgical plane, effectively separating the lesion from the adjacent normal frontal lobe structures.

**Figure 6:**
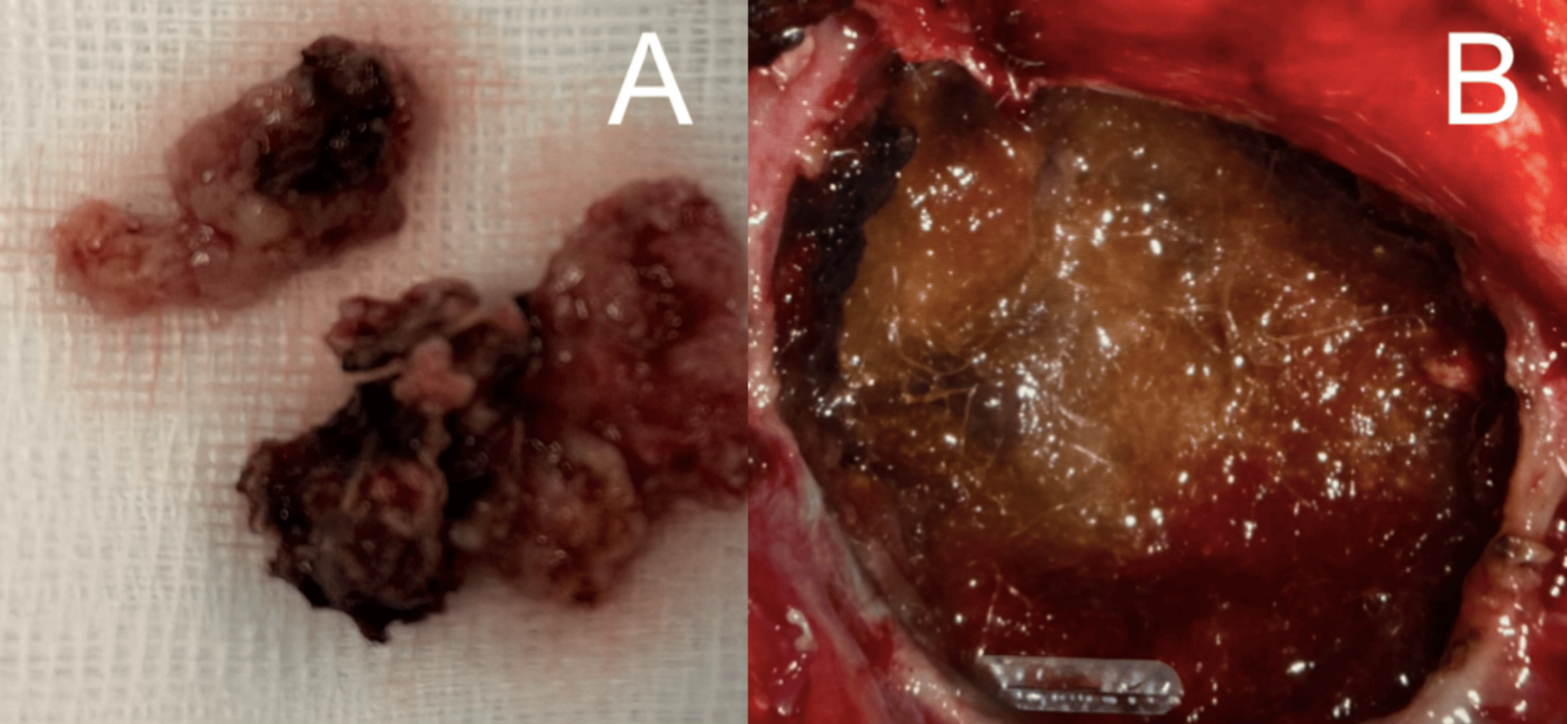
Gross specimen and surgical cavity (A) Complete tumor specimen, corresponding to frontal convexity meningioma. The lesion demonstrates a solid consistency and superficial vascularization, with macroscopic features consistent with a WHO grade I diagnosis. (B) Surgical cavity confirming the total tumor removal with minimal brain exposure. WHO: World Health Organization

**Figure 7:**
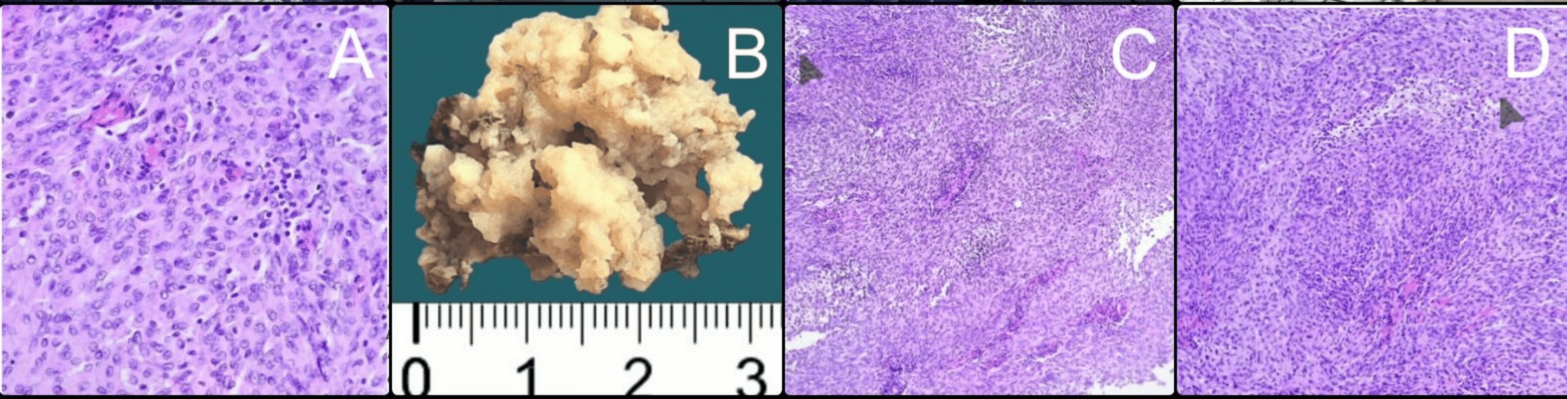
Histopathological features of fibrous meningioma (A, C, and D) Photomicrographs demonstrating spindle-shaped tumor cells arranged in fascicles, with characteristic meningothelial whorl formation and scattered psammoma bodies, consistent with fibrous meningioma. (B) Gross pathological specimen showing a well-circumscribed, firm lesion. Overall findings were consistent with WHO grade I fibrous meningioma. WHO: World Health Organization

Progressive devascularization of the lesion was subsequently performed. Intraoperatively, the tumor appeared firm, well-demarcated, and encapsulated, with prominent venous drainage into the superior sagittal sinus, which was meticulously preserved throughout the arachnoid dissection. The arterial pedicles supplying the tumor base were sequentially identified and divided, effectively reducing the arterial vascular supply and substantially minimizing intraoperative bleeding. This allowed complete isolation of the lesion, facilitating its mobilization and en bloc resection (Figure 6). Complete tumor resection was achieved intraoperatively, and the diagnosis of World Health Organization (WHO) grade I fibrous meningioma was subsequently confirmed by histopathological analysis (Figure 7).

**Figure 8:**
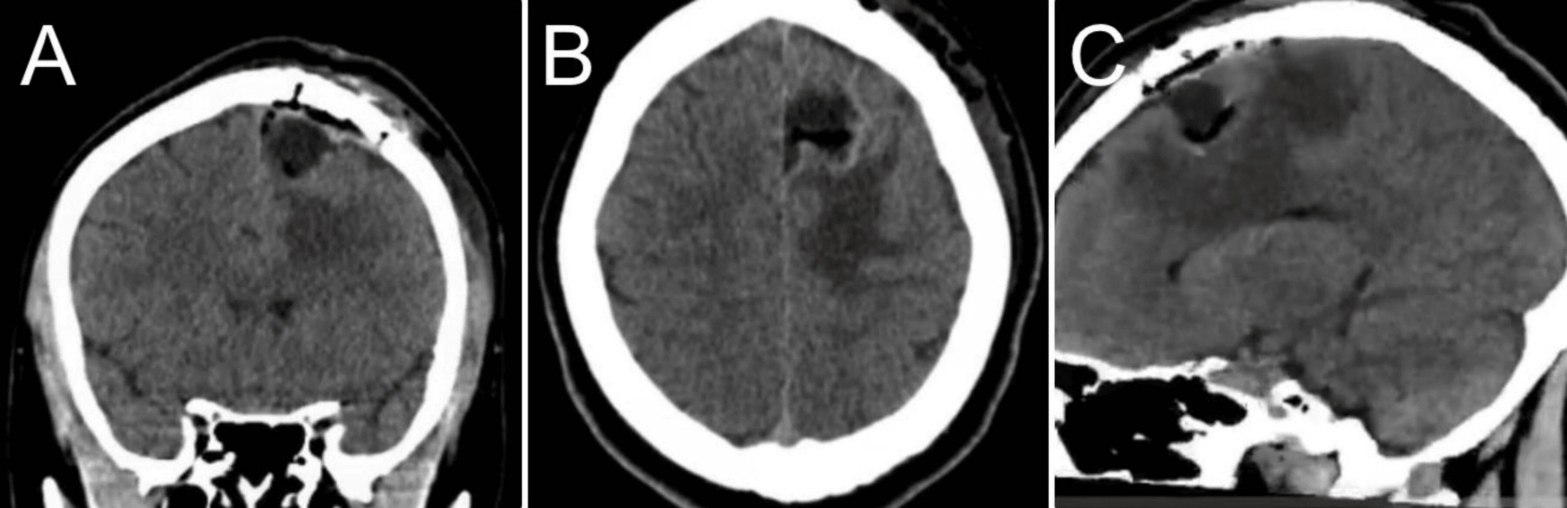
Postoperative CT after tumor resection (A) Coronal, (B) axial, and (C) sagittal postoperative CT images demonstrating the surgical cavity after tumor resection. Confirming complete tumor resection without evidence of structural anatomical damage and a minimal frontal craniotomy with very minimal brain exposure. CT: computed tomography

Careful circumferential dissection was performed under continuous magnification using BHS Technologies RoboticScope exoscope, allowing optimal visualization with dynamic adjustment of focus, illumination, and zoom, while preserving adjacent important buenostructures. The lesion was progressively mobilized and ultimately resected in its entirety, achieving a gross total resection (Figure 8).

## Discussion

Minimally invasive robotic-assisted surgery has progressively transformed the surgical management of intracranial tumors, particularly frontal meningiomas located within the anterior cranial fossa and frontal convexity. The combination of robotic technology, neuronavigation, endoscopic visualization, and keyhole craniotomy techniques provides a modern surgical strategy focused on maximizing tumor resection while minimizing collateral tissue injury [11-14]. In the present case, robotic-assisted minimally invasive frontal surgery allowed successful tumor removal with excellent postoperative neurological recovery and reduced surgical morbidity.

Conventional transcranial approaches for frontal meningiomas frequently require wide craniotomies, extensive soft tissue dissection, and significant frontal lobe retraction, all of which may contribute to postoperative edema, cosmetic deformities, prolonged hospitalization, and neurological deficits [11,12]. Minimally invasive supraorbital and eyebrow approaches were developed to reduce these complications while maintaining adequate surgical exposure. Several contemporary studies have demonstrated that minimally invasive approaches achieve gross total resection rates comparable to traditional craniotomies with reduced perioperative morbidity [13,14].

The supraorbital keyhole approach has become one of the most commonly utilized minimally invasive techniques for anterior skull base meningiomas. Reisch and Perneczky initially described the advantages of this corridor, emphasizing reduced brain manipulation, improved cosmetic outcomes, and shorter recovery periods [15]. Subsequent anatomical and clinical studies confirmed that the supraorbital route provides adequate exposure for selected frontal and parasellar lesions while preserving neurovascular structures [16]. Endoscopic assistance further enhances visualization within narrow operative corridors and improves illumination of deep anatomical regions that are otherwise difficult to access using conventional microscopy alone [17].

Robotic integration into minimally invasive cranial surgery represents a significant technological evolution. Robotic systems provide improved instrument stability, tremor filtration, enhanced precision, and ergonomic advantages during microsurgical dissection [18]. These characteristics are particularly valuable when operating within restricted surgical corridors adjacent to eloquent cortical areas and critical vascular anatomy. Additionally, robotic-assisted platforms facilitate integration with advanced neuronavigation systems, allowing precise preoperative trajectory planning and real-time intraoperative orientation [19].

Another major advantage of minimally invasive robotic surgery is its positive impact on postoperative recovery and patient quality of life. Reduced muscle dissection, smaller skin incisions, and limited craniotomy size contribute to diminished postoperative pain, faster mobilization, shorter hospital stays, and improved cosmetic satisfaction [20]. Early rehabilitation and rapid reintegration into daily activities may be particularly beneficial in younger patients and individuals with high functional demands.

From an oncological perspective, careful patient selection remains essential. While minimally invasive approaches are highly effective for small- to medium-sized frontal meningiomas without extensive vascular encasement or diffuse skull base invasion, larger lesions with significant edema, multicompartment extension, or major vascular involvement may still require conventional open approaches [21]. Therefore, individualized surgical planning based on tumor location, size, vascularity, and relationship to eloquent structures remains critical.

The learning curve associated with robotic-assisted neurosurgery also represents an important consideration. Successful implementation requires specialized surgical training, multidisciplinary collaboration, familiarity with advanced technological platforms, and institutional infrastructure capable of supporting robotic procedures [22]. Furthermore, economic limitations remain a challenge, particularly in low- and middle-income healthcare systems where access to robotic platforms may be restricted. Despite these barriers, continued technological development and increasing surgical experience are likely to improve accessibility and reduce implementation costs over time.

Future innovations in robotic neurosurgery may substantially expand the role of minimally invasive cranial surgery. Emerging technologies such as augmented reality-guided neuronavigation, artificial intelligence-assisted surgical planning, flexible robotic instrumentation, and haptic feedback systems could further improve surgical precision and safety [23]. These developments may ultimately enhance tumor resection while minimizing neurological morbidity and improving long-term patient outcomes.

## Conclusions

Minimally invasive robotic-assisted surgery is a safe and effective alternative for the treatment of selected frontal meningiomas. The combination of robotic technology, neuronavigation, and keyhole approaches allows adequate tumor resection while reducing brain manipulation, surgical morbidity, postoperative pain, and recovery time.

Compared with conventional transcranial approaches, robotic-assisted minimally invasive surgery may provide improved surgical precision, shorter hospitalization, faster rehabilitation, and better cosmetic outcomes. Careful patient selection remains essential to optimize surgical and oncological results. Although limitations such as cost, technological availability, and the surgical learning curve persist, continued advances in robotic neurosurgery are expected to further expand its role in cranial tumor surgery.
